# Magma Degassing as a Source of Long‐Term Seismicity at Volcanoes: The Ischia Island (Italy) Case

**DOI:** 10.1029/2019GL085371

**Published:** 2019-12-23

**Authors:** E. Trasatti, V. Acocella, M. A. Di Vito, C. Del Gaudio, G. Weber, I. Aquino, S. Caliro, G. Chiodini, S. de Vita, C. Ricco, L. Caricchi

**Affiliations:** ^1^ Istituto Nazionale di Geofisica e Vulcanologia Italy; ^2^ Department of Earth Sciences Università degli Studi di Roma Tre Rome Italy; ^3^ Department of Earth Sciences University of Geneva Switzerland

**Keywords:** degassing, geodetic data, inverse modelling, magmatic source, physics of volcanism, resurgence

## Abstract

Transient seismicity at active volcanoes poses a significant risk in addition to eruptive activity. This risk is powered by the common belief that volcanic seismicity cannot be forecast, even on a long term. Here we investigate the nature of volcanic seismicity to try to improve our forecasting capacity. To this aim, we consider Ischia volcano (Italy), which suffered similar earthquakes along its uplifted resurgent block. We show that this seismicity marks an acceleration of decades‐long subsidence of the resurgent block, driven by degassing of magma that previously produced the uplift, a process not observed at other volcanoes. Degassing will continue for hundreds to thousands of years, causing protracted seismicity and will likely be accompanied by moderate and damaging earthquakes. The possibility to constrain the future duration of seismicity at Ischia indicates that our capacity to forecast earthquakes might be enhanced when seismic activity results from long‐term magmatic processes, such as degassing

## Key Points

- About 30 years of leveling data show constant rate subsidence of the resurgent block at Ischia (Italy)
- Mechanical and thermo‐petrological simulations indicate magmatic degassing as main cause of the subsidence of the resurgent block
- Seismicity marks an acceleration of decades‐long subsidence, driven by degassing that will continue for hundreds/thousands years

## 1 Introduction

**Figure 1:**
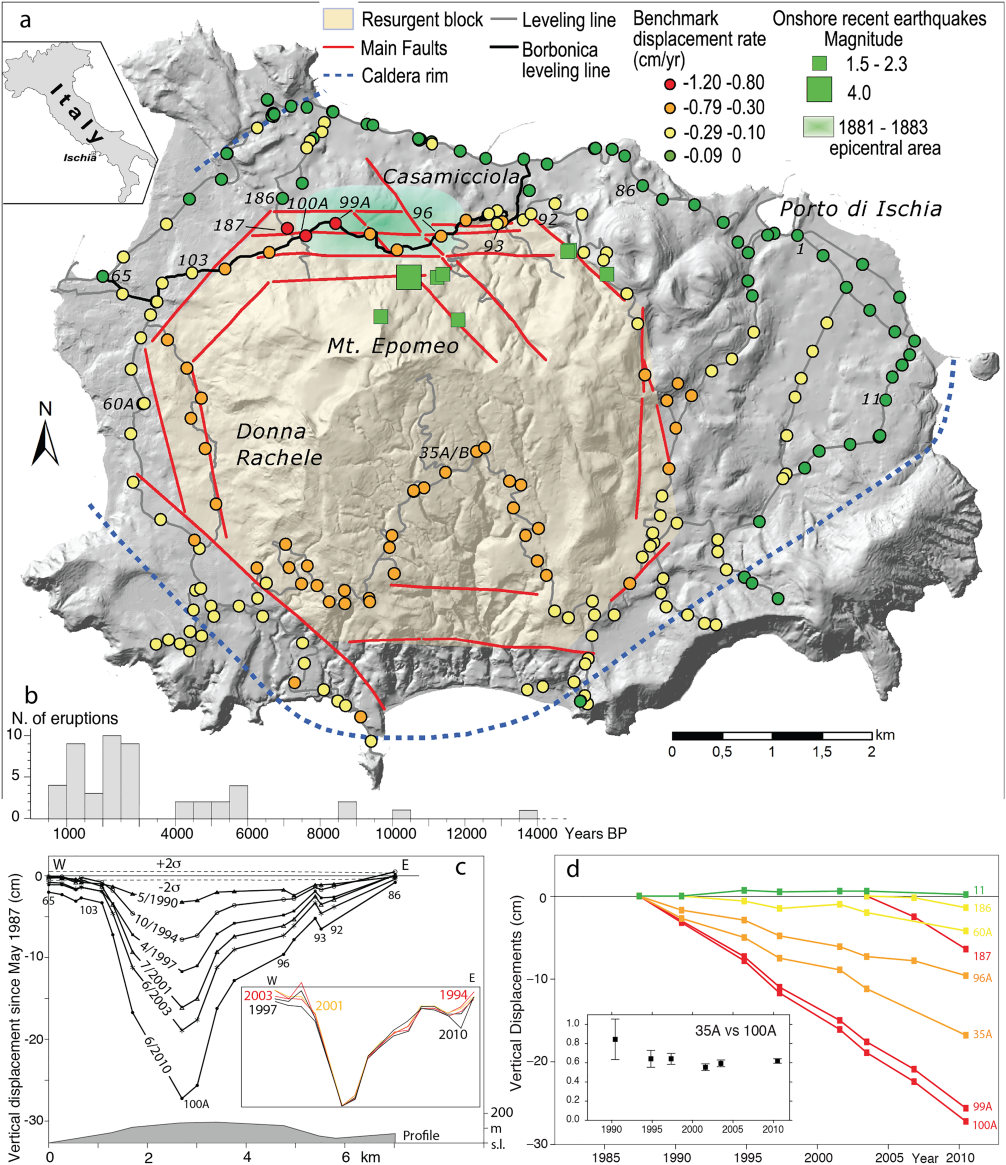
Ischia island, eruption history and leveling data. (a) Structural sketch map reporting the main faults, the resurgent block, the leveling benchmarks (some identified with numbers), the onshore recent earthquakes (from 1999 to 2017), and the 1881–1883 epicentral area. (b) Frequency of eruptions in the last 15 ka (de Vita et al., 2010; Sbrana et al., 2018 and references therein). (c) Leveling data along the “Borbonica measurement line” (thick line in panel a) between 1987 and 2010; in the inset their normalized values. (d) Time‐series of selected benchmarks, showing different amounts of subsidence. In the inset the ratio between leveling changes at benchmarks 35A and 100A is reported.

Long‐term seismic forecast usually assumes constant stress accumulation resulting from regional tectonic processes. This approach is hindered in volcanic areas, where local stress fields from magmatic processes interact with regional structures, inducing transient seismicity, often with apparently random space‐time patterns. Therefore, long‐term seismic forecast in volcanic areas has been extremely challenging so far (White & McCausland, 2019 and references therein). Here we show how identifying recurrent earthquake patterns in volcanic areas provides clues to understand the role of magmatism on seismicity and improve long‐term forecasting. For this, we consider the recent deformation of Ischia volcano (Italy) and relate it to the seismicity of the last centuries. Ischia experienced one of the highest uplift measured at any volcano, reaching ~1,000 m in 35–55 ka (de Vita et al., 2010; Sbrana et al., 2018 and references therein). This process, called resurgence, produced a fault‐bounded block (Figure 1a), with topmost uplift to the NW and an overall tilt downward SE (Acocella & Funiciello, 1999). Volcanic activity during resurgence alternated with variably lasting periods of quiescence. The last phase of activity started at ~10 ka BP (Figure 1b) and peaked in the last ~6 ka, with tens of eruptions around the resurgent block, the last in 1302 AD (de Vita et al., 2010). The faults along the northern boundary of the block (Casamicciola area, Figure 1a) triggered shallow (<2 km depth) and destructive earthquakes, in 1228 (700 casualties), 1796 (7 casualties), 1828 (30 casualties), 1881 (estimated magnitude 4.4–4.7; >120 casualties), 1883 (estimated magnitude 4.3–5.2; >2300 casualties), and 2017 (M = 4.0; 2 casualties) (Cubellis & Luongo, 1998; D'Auria et al., 2018; De Novellis et al., 2018; Nappi et al., 2018; Selva et al., 2019). The similarities among these events (fault location, shallow depth subvertical geometry, magnitude between 4 and 5, and dip‐slip motion) provide favorable conditions to investigate their origin and to attempt to forecast future events. For this, we use geologic and geodetic data, along with mechanical and thermal‐petrological models.

## 2 Surface Deformation at Ischia Since 1987

The vertical ground deformation of Ischia has been monitored by means of a dense leveling network established by Istituto Geografico Militare in 1913. This network, implemented through time, is 110 km long and includes 260 benchmarks. The reference benchmark lies on the northeastern coast, at Porto di Ischia (1 in Figure 1a; Church of St. Maria Portosalvo, elevation = 3.0360 m). The network is mainly arranged in loops, to minimize and check the errors associated with the measurements. The orthometric height of all benchmarks, computed with respect to the reference site, is obtained by minimizing the misclosures of each loop by means of a least squares compensation applied to the measured elevation differences between successive benchmarks (Del Gaudio et al., 2011). Measurements were carried out in 1978, 1984, 1987, 1990, 1994, 1997, 2001, 2003, 2006**,** and 2010 (Table S1 reports the original measurements since 1984). Here we select the more complete time series since 1987. The 1984 dataset is not considered because of its limited extent.

The vertical velocity of the leveling benchmarks of eight campaigns between 1987 and 2010 (Figure 1a) reveals four subsidence categories: (i) negligible (−0.1 to 0 cm/yr); (ii) minor (−0.3 to −0.1 cm/yr); (iii) moderate (−0.3 to −0.8 cm/yr); and (iv) major (−0.8 to −1.2 cm/yr) with related uncertainty of ~0.02 cm/yr. Minor or no subsidence occurs along the coast; minor subsidence is found around the resurgent block; moderate to high subsidence occurs within the block, especially in its most uplifted NW boundary. The subsidence on the NW boundary focuses along the ~E‐W trending “Borbonica measurement line” (reaching 27 cm at benchmark 100A; Figure 1). Within the block, the subsidence area is wider and drops to 17 cm (benchmark 35A; Figures 1a and 1d). A similar subsidence, focused in the NW border of the block and south of Mt Epomeo, was observed by InSAR data (Castaldo et al., 2017). Subsidence throughout the island develops linearly over 23 years (Figure 1d). This underlies the self‐similarity of the normalized displacement pattern between 1987 and 2010 (Figure 1c, inset) and suggests the activity of the same source/s. Furthermore, the two distinct minima (benchmarks 100A and 35A) show a stable ratio through time, indicating interdependence in the process/es controlling the deformation (Figure 1d, inset).

## 3 Mechanical Model and Thermal‐Petrological Simulations

### 3.1 Mechanical Model of Geodetic Data

Because of the larger‐ and smaller‐scale patterns of the measured deformation, we consider two analytical models representing two deformation sources, a sill‐like source (Fialko et al., 2001) that mimics the island‐scale subsidence and a fault (Okada, 1992) matching the short‐scale but intense deformation at the NW margin of the block. These sources represent the simplest and most conservative conditions to model the observed deformation. The two analytical models (fault and sill) are jointly inverted in elastic regime, which is supported by the linear trend of the time‐series (Figure 1d). We use the VSM – Volcano and Seismic source Modelling – tool (Trasatti, 2019), whose core optimization code minimizes a chi‐square function. The global optimization by means of the VSM tool is followed by a second step based on Bayesian inference on the generated ensemble of models, to retrieve the Posterior Probability Density (PPD) functions and the mean values of the unknowns (Sambridge, 1999). To limit the inverted parameters, the fault is set to be dip‐slip, east‐west striking, and southward dipping at 80°, in accordance with seismology studies on the 2017 event (D'Auria et al., 2018; De Novellis et al., 2018). It extends from easting 405.5 km (UTM projection, zone 33) and is 500 m wide. The PPD functions associated with the 9 unknowns are reported in the Figures S1 and S2. The volume variation of the sill is computed as

(1) $$\Delta V=\frac{8}{3}\left(1-\nu \right)r^{3}\frac{\Delta P}{\mu },$$

where the Poisson coefficient ν = 0.25, *r* is the radius, Δ*P* is the overpressure, and *μ* is the rigidity (Amoruso et al., 2014). The factor Δ*P*/*μ* is often indicated as “potency,” and it is inverted as a single parameter. The best‐fit results are reported in Table S2.

Finally, each leveling campaign since 1990 is considered by relative quote variations with respect to 1987. Seven elevation changes datasets are obtained, related to the following periods: 1987–1990, 1987–1994, 1987–1997, 1987–2001, 1987–2003, 1987–2006, and 1987–2010. Each inversion retrieves the potency of the sill and the fault slip, while their geometry and position are unchanged.

**Figure 2:**
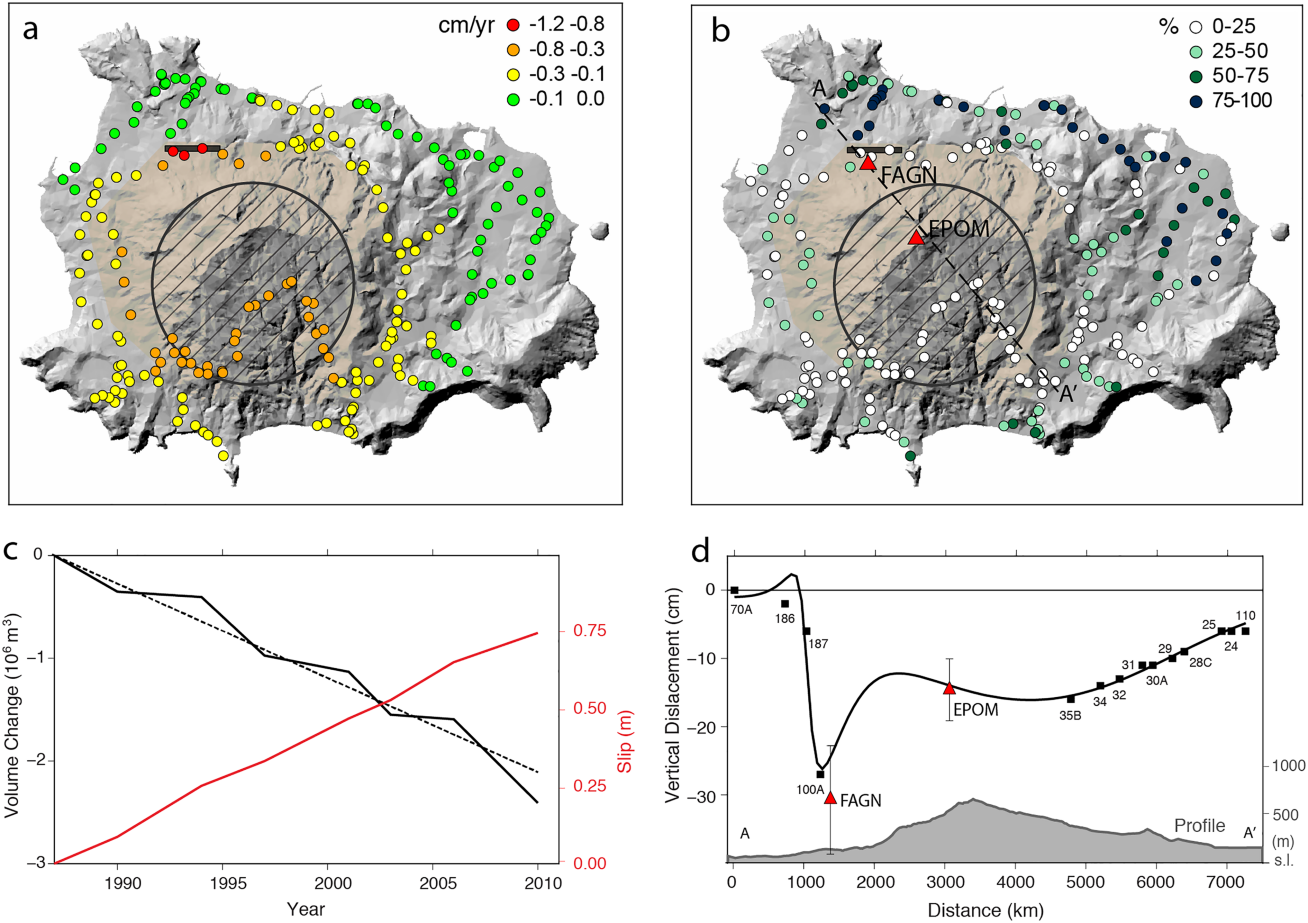
Geodetic inversion results. (a) Modelled leveling data and (b) percentual residuals between observed (Figure 1a) and modelled data. The red triangles in (b) are the GPS stations along the A‐A′ profile. The surface projections of the sill and the fault are reported in black, while the resurgent area is in transparent light brown in (a)‐(b). (c) Volume change at the sill (black) and dip‐slip at the creeping fault (red) versus time. The dashed black line is the average volume change rate. (d) Displacements along the NW‐SE‐oriented A‐A′ profile as depicted in (c). The black squares are the leveling benchmarks, and the red triangles are the GPS (1997‐2003).

Results from the optimization of the leveling data and Bayesian inference (Table S2; Figures 2, S1, and S2) suggest that the subsidence, modelled by a deflating sill‐like body, can be interpreted as a magmatic reservoir centered below the resurgent block at ~2.2 km depth, contracting at 1.05 ± 0.07 × 10^5^ m^3^/yr. An additional local component of subsidence is associated with the aseismic dip‐slip (slip‐rate of 3.1 cm/yr) of an ~E‐W trending southward dipping fault located in the NW boundary of the block, with area of 0.46 km^2^ extending from near surface (Figures 2a and 2b). Figure 2c reports the trends of the volume variation of the sill and the dip‐slip at the fault. Both the sill deflation rate and fault creep rate are constant over the measurement period, producing a cumulative volume decrease of 2.4 × 10^6^ m^3^ and a total slip of 75 cm. Their combined surface displacement along a NW‐SE profile matches both our leveling data and GPS vertical velocities 1997−2003 (Del Gaudio et al., 2011), confirming that subsidence culminates along the faults bordering the previously most uplifted NW part of the resurgent block (Figure 2d).

### 3.2 Coupled Thermo‐Petrological Simulations

We use the thermodynamic software rhyolite‐MELTS (Gualda et al., 2012) to calculate the volumetric variations associated with cooling, crystallization, and release of volatiles of a trachytic magma that is representative of compositions erupted at Ischia (Poli et al., 1987). The petrological data are coupled with thermal models simulating the temporal evolution of temperature in an instantaneously emplaced cylindrical magma body. We solve the two‐dimensional heat conduction equation in axisymmetric formulation, which can be written as

(2) $$\mathrm{\rho c}\frac{\mathrm{\partial T}}{\mathrm{\partial t}}=\frac{1}{r}\frac{\partial }{\mathrm{\partial r}}\left(\mathrm{rk}\frac{\mathrm{\partial T}}{\mathrm{\partial r}}\right)+\frac{\partial }{\mathrm{\partial z}}\left(k\frac{\mathrm{\partial T}}{\mathrm{\partial z}}\right)+\mathrm{\rho L}\frac{\partial X_{c}}{\mathrm{\partial t}},$$

where T is the temperature, t is the time, r is the radial coordinate relative to the symmetry axis, k is the thermal conductivity, z is the depth, ρ is the density, L is the latent heat of crystallization, c is the specific heat, and X_c_ is the crystal fraction. An explicit finite difference method is used to discretize the heat equation on a numerical mesh with dimensions of 300 × 300 computational cells, corresponding to a physical domain of 5 × 5 km. To implement latent heat of crystallization, we use the relationship between crystal fraction (X_c_) and temperature:

(3) $$X_{c}=1-\left(\frac{1}{1+e^{\left(\frac{890-T}{14}\right)}}\right),$$

where T is the temperature in °C. This relation is chosen in order to fit the variation of crystal content and temperature for shallow crystallization of Ischia trachyte magma as calculated from rhyolite‐MELTS. All thermal modelling is performed with the following physical parameters: initial geothermal gradient 30°C km^−1^, latent heat of fusion 3.13 × 10^5^ J kg^−1^, specific heat 1,000 J Kg^−1^ K^−1^, and density of 2,700 kg m^−3^. A temperature‐dependent thermal conductivity is implemented in all runs using the values for average crust presented in (Whittington et al., 2009). In line with existing estimates (Galetto et al., 2017), we consider a 2–4 km deep sill‐like intrusion with aspect ratio (height/diameter) between 0.2 and 0.4 and initial volume between 3 and 15 km^3^. The initial H_2_O and CO_2_ content of the melt is fixed to 2.44 wt. % and 0.022 wt. %, respectively, which is comparable to the volatile content measured in melt inclusions (Moretti et al., 2013). Simulations are performed at a pressure of 75 MPa. In each numerical simulation, magma is injected at its liquidus temperature of 1025°C and allowed to cool down to its solidus at 825°C. The sensitivity of results to the initial geothermal gradient was tested by running models with 20, 30, or 40°C km^−1^, which gives overall similar results (Figure S3). Fixed temperature constraints are imposed on the upper (i.e., 8°C at surface) and lower boundaries of the model volume determined by the geothermal gradient. Zero flux boundary conditions are implemented on the left and right sides of the model. Advection of heat (e.g., due to eruptions and/or hydrothermal circulation) is neglected. As both phenomena contribute to increase the cooling rate of Ischia's shallow magmatic reservoir over the timescale studied here, we consider our calculated volumetric contraction rates to be minimum estimates.

**Figure 3:**
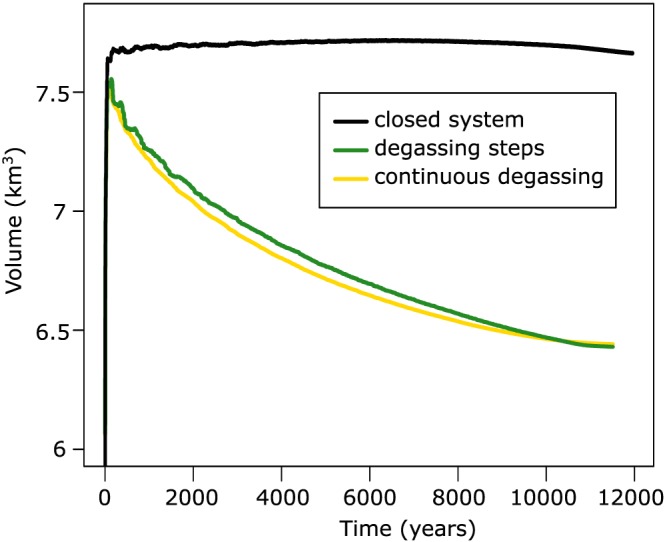
Temporal evolution of volume for different degassing scenarios (closed system, black; open system, green and yellow).

The volume of magma within each temperature interval of 25°C is tracked in time during cooling using the thermal model. Summing the volumes calculated for each temperature interval within the intrusion (and above solidus temperature) yields the evolution of the volume of the intrusion in time. Three scenarios are considered for volumetric changes within the intrusions: closed system crystallization, stepwise degassing, and continuous degassing (Figure 3). In closed system conditions, the volume variations are associated with crystallization and the release of excess volatiles. Initially, the volume of the intrusions increases because of the release of excess volatiles; however, at near solidus conditions crystallization overwhelms bubble expansion and the volume decreases. Such near solidus decrease of volume was never sufficiently large to account for the observed deflation rates and would imply lack of magmatic fluid release at the surface, which is in contradiction with the fumarolic manifestations of Ischia (Tedesco, 1997); thus, this scenario was not considered further. In the open system case, all volatiles are removed instantaneously once exsolved from the melt, which leads to continuous subsidence of the system (yellow curve; Figure 3). In the open system with stepwise degassing (green curve), the excess fluid is residing in each parcel of cooling magma until a critical bubble volume fraction 30% relative to the melt and crystals (percolation threshold) is reached upon which degassing occurs. In the stepwise degassing scenario, degassing is modelled to start once the magma cooled below 850°C. The second and third degassing steps are modelled once the magma cools below 825°C and 800°C. In both cases of the open system the rate of volume contraction is larger during the younger history of the intrusion as the cooling rate, and therefore, the rate of degassing, is faster. We consider here only the open system case, as the two scenarios gave similar results and it does not require to implement a percolation threshold of uncertain value.

**Figure 4:**
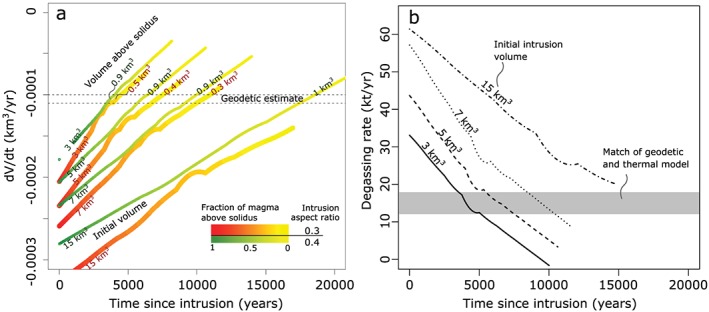
Thermal‐petrological simulations. (a) Rate of volume change, dV/dt, versus time since emplacement of degassing magma bodies for different initial volumes. Color coding indicates magma above solidus in time for intrusion aspect ratios of 0.3 and 0.4. Dashed lines show dV/dt as calculated by geodetic inversion. Upper labels on the curves indicate the volume of magma above solidus at the interception of the thermal‐petrological and geodetically estimated rate. (b) Degassing rate versus time since intrusion computed for magma bodies of different initial volume. Grey bar shows the match between thermal and geodetic estimates.

The maximum contraction rate produced considering exclusively magma crystallization is ~3.15 × 10^4^ m^3^/yr, one order of magnitude smaller than the measured values. As cooling and crystallization of a fluid saturated magma lead to accumulation of excess fluids, we calculate the combined effect of crystallization and continuous degassing. The resulting rate of volumetric contraction decreases with time for a wide range of initial sill volumes (Figure 4a). Furthermore, the deflation rates match the measured value at increasing times since the onset of magma injection. The eruptive record suggests that the current magmatic cycle started ~6 ka ago (Figure 1b). We find that the continuous release of excess fluids of a sill with initial volume of ~5 km^3^ injected at 6 ka best matches the currently measured deformation rate and it is sufficiently large to maintain eruptible magma during the period of volcanic activity of Ischia (Figures 1c and 4a). Our calculations agree with measured deflation rates for sill with aspect ratio between 0.3 and 0.4, while magma bodies of smaller aspect ratio (0.2) produce deformation rates that are smaller than the measured ones (Figure S4).

## 4 Discussion and Conclusions

Isotopic measurements of the fumaroles confirm the contribution of magmatic fluids to the released volatiles. At Donna Rachele fumaroles, ^3^He/^4^He ratios range from 3.25 to 3.95 R/Ra, where R/Ra is the ratio between the fumarolic ^3^He/^4^He and that of air (Tedesco, 1997), higher than those from nearby Campi Flegrei and Vesuvius volcanoes. Degassing rates calculated with our models agree also with stable isotopes measurements of thermal spring waters and fumaroles, in which a magmatic component cannot be detected (Caliro et al., 1999; Chiodini et al., 2004; Di Napoli et al., 2009; Giggenbach, 1992; Panichi et al., 1992; Figure S5 and Table S3). In fact, the rate of fluid release required to explain the subsidence is 12–18 kt/yr (Figure 4b), a negligible amount with respect to the emission rates at the Donna Rachele fumaroles alone (~500 kt/yr of condensed meteoric steam; Chiodini et al., 2004; Figure 1 and Table S3). The flux of deeply derived CO_2_ measured at Donna Rachele (~3 kt/yr; Chiodini et al., 2004) is orders of magnitude lower than that affecting the nearby volcanoes Campi Flegrei (400–1000 kt/yr; Cardellini et al., 2017) and Vesuvius (~55 kt/yr; Frondini et al., 2004), in agreement with our model of intrusion that has already degassed most of the fluids.

**Figure 5:**
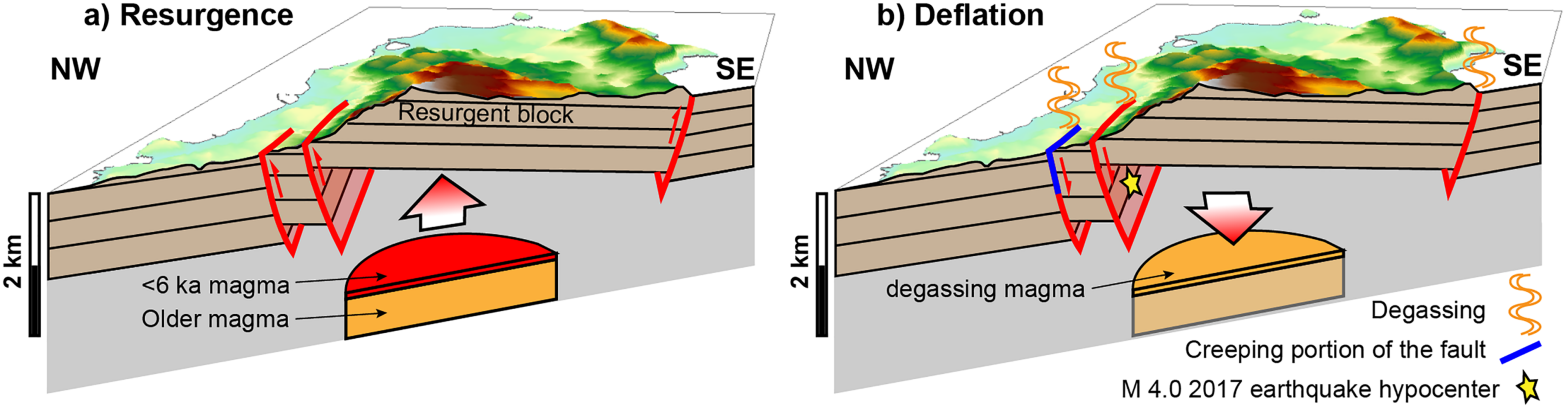
The proposed model along a NW‐SE section. (a) Resurgence produces a tilted uplifted block, activating inward dipping reverse faults. (b) The degassing of the magma emplaced in the last ~6 ka (red sill in a) deflates the previously uplifted block, reactivating the faults to the NW with an extensional motion. The reactivation produces seismicity (yellow star) and creep (shallow blue segment of fault).

Magma degassing from a cooling magma body is responsible for subsidence at Ischia (Figure 5). The normal focal mechanism of the 2017 Casamicciola earthquake (De Novellis et al., 2018; Nappi et al., 2018) suggests that seismicity is consistent with a deflationary forcing process. As the 1881–1883 and previous Casamicciola earthquakes are consistent in location and kinematics with the 2017 event (Cubellis & Luongo, 1998; D'Auria et al., 2018), deflation may has been continuous for at least centuries. Additionally, deflation matches in location, asymmetry, and fault activation the longer‐term resurgence uplift, albeit with inverted kinematics (Figure 5). This supports the magmatic nature of the modelled source. Therefore, Ischia is a unique case of deflating resurgence accompanied by recurrent seismicity.

The implications for seismic hazard are twofold. First, seismicity at Casamicciola peaked with two consecutive earthquakes in 1881 and 1883. Even though their inferred epicenters overlap within uncertainties (Cubellis & Luongo, 1998), their sequential occurrence suggests the slip of two fault segments, possibly one triggering the other. This possibility should be considered carefully after the 2017 earthquake, as this event might promote slip on nearby faults, as the modelled creeping fault, which has been building up an equivalent magnitude 4.6 in 23 years (Kanamori, 1977), using a mean shear modulus of 10 GPa. This structure seems parallel to and partially overlapping the 2017 seismogenic fault and seismic slip of its deeper locked portion cannot be excluded, possibly loaded by the creep of its upper section and slip during the 2017 event. Coulomb stress models (Harris, 1998; Figure S6) suggest that 23 years of creep have accumulated a stress of ~0.1 MPa at the fault base. This should be a minimum estimate, as the fault has been certainly creeping for longer. Second, our results also allow us to estimate whether and for how long will the recurrent seismicity on the northern border of the block continue. The significant increase in eruptive activity suggests that magma was emplaced at ~6 ka (Figure 1c; de Vita et al., 2006; de Vita et al., 2010). The thermal models suggest that, independent of the exact timing and initial intrusion volume (3–15 km^3^), to date at least 0.4 to 0.9 km^3^ of magma are still cooling and degassing (Figure 4a). Hence, the modelling shows that there is enough volume in the magma chamber to sustain degassing and subsidence will continue for hundreds to thousands of years at least (i.e., the time required for magma cooling to its solidus temperature) or until any future intrusion. This is a minimum time estimate, as it considers that no additional magma injection occurred since 6000 years ago. Our results suggest that degassing activity will continue and will be accompanied by future earthquakes near Casamicciola, calling for immediate mitigation measures.

The case of Ischia finds wider application to similar calderas with resurgent block, whose boundary focuses deformation and seismicity, as Pantelleria (Italy), similarly subsiding because of degassing (Mattia et al., 2007) and Sierra Negra and Alcedo (Galapagos), recently both experiencing net uplift (Chadwick et al., 2006; Galetto et al., 2019). The case of Ischia shows how the controlling mechanisms of seismicity in volcanic areas may be identified. This implies that our capacity of forecasting seismic activity might be significantly enhanced in regions where earthquakes are associated with long‐term magmatic processes.

## Supporting information

Supporting Information S1

Data Set S1

## Data Availability

Data access information and additional methodologies are provided in the manuscript or in the supplementary information. Leveling data are also available at https://pangaea.de/10.1594/PANGAEA.909710.
