# Supplementary material for: Magma Degassing as a Source of Long‐Term Seismicity at Volcanoes: The Ischia Island (Italy) Case
Source: Geophys Res Lett. 2019 Dec 23;46(24):14421–9. doi: 10.1029/2019GL085371 (PMC7043361; doi:10.1029/2019GL085371)
Supplement: Supplementary file 2 — Data Set S1 [file GRL-46-14421-s002.docx]

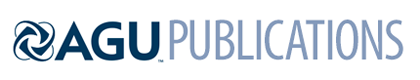


*Geophysical Research Letters*

Supporting Information for

**Magma degassing as a source of long-term seismicity at volcanoes**

E. Trasatti^1^, V. Acocella^2^, M.A. Di Vito^1^, C. Del Gaudio^1^, G. Weber^3^, I. Aquino^1^, S. Caliro^1^, G. Chiodini^1^, S. de Vita^1^, C. Ricco^1^, L. Caricchi^3^

^1^Istituto Nazionale di Geofisica e Vulcanologia, Italy.

^2^Università degli Studi di Roma Tre, Rome, Italy.

^3^Univerisity of Geneva, Switzerland.

**Contents of this file**

Figures S1 to S6

Tables S1 to S3

**Additional Supporting Information (Files uploaded separately)**

Table S1. Altimetric data and relative errors of the leveling measurements carried out between 1984 and 2010. (xls file).


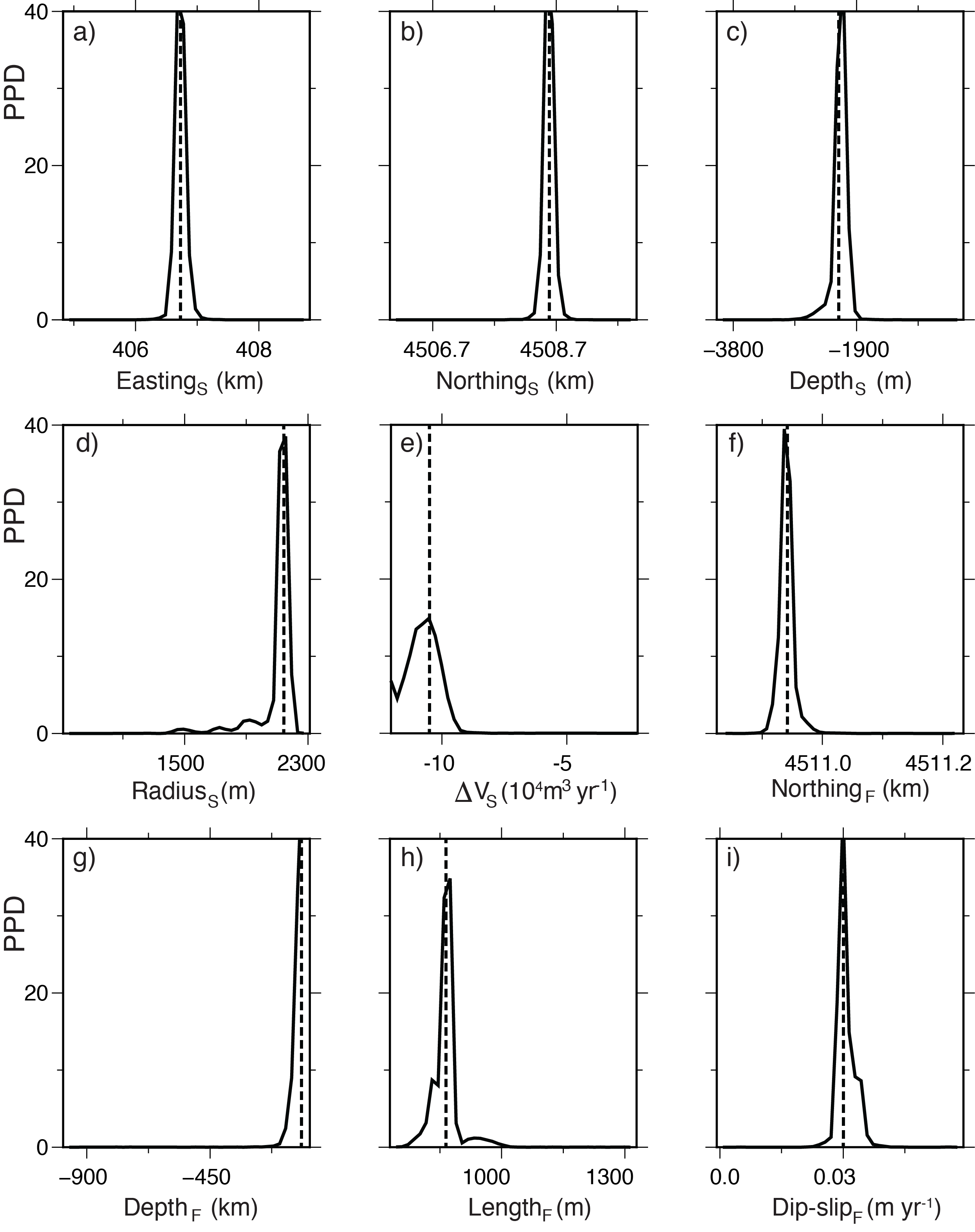


Figure S1. 1D PPD functions of the inverted parameters. Results from the Bayesian inference on the generated ensemble of models. Subscript S is referred to the parameters of the sill-like source, F to those of the fault. The vertical dashed line is the mean model, i.e., the best representative of the most probable set of models, as reported in Table S2. Easting and Northing are in UTM projection (zone 33). The volume variation in (e) is a parameter derived from the radius and potency of the sill (see methods).


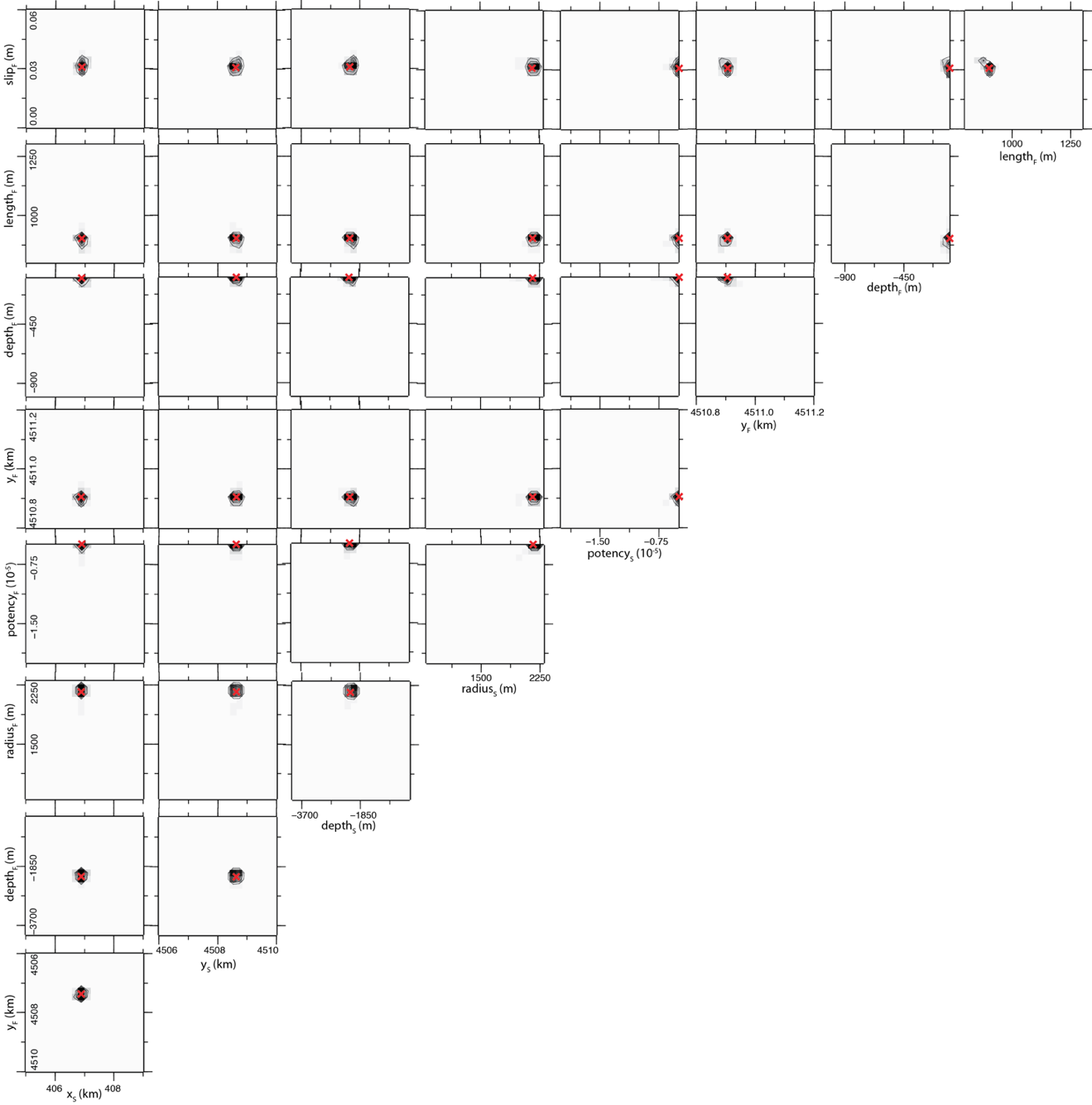


**Figure S2**. 2D marginal PPD functions for the inverted parameters. Results from the Bayesian inference on the generated ensemble of models. Contour every 10% confidence. The red cross is the mean model. Subscript S is referred to the parameters of the sill-like source, F to those of the fault.


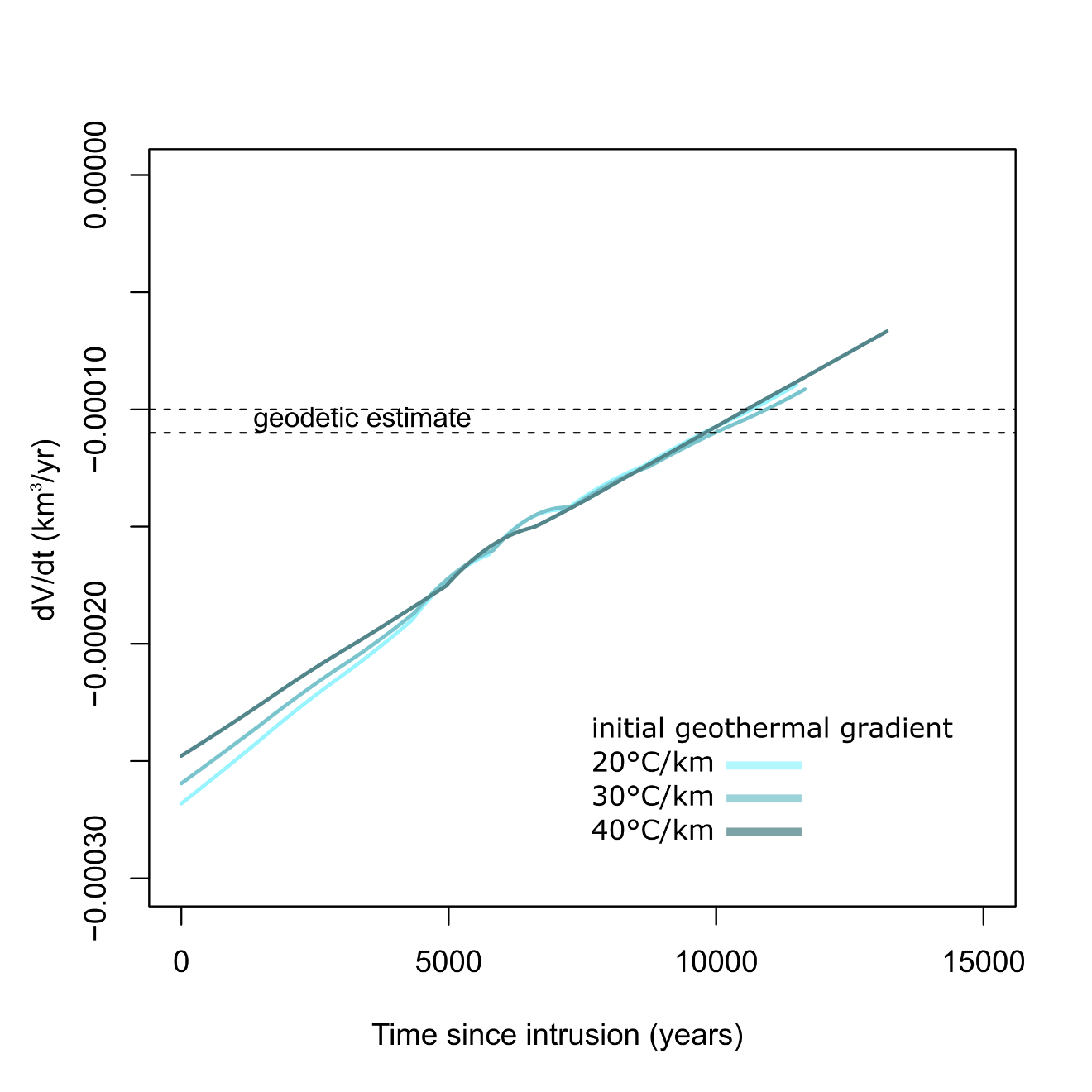


**Figure S3.** Impact of initial geothermal gradient on rate of volumetric change. The initial intrusion volume is 7.6 km^3^ with aspect ratio of ~0.3 emplaced at a depth of 2.5 km. Models were run with initial geothermal gradients of 20°C km^-1^ (cyan curve), 30°C km^-1^ (blue curve) and 40°C km^-1^ (dark blue curve). The resulting volumetric change rates for the three setups match the geodetically constrained volumetric deflation rate at overall similar time after the onset of intrusion.


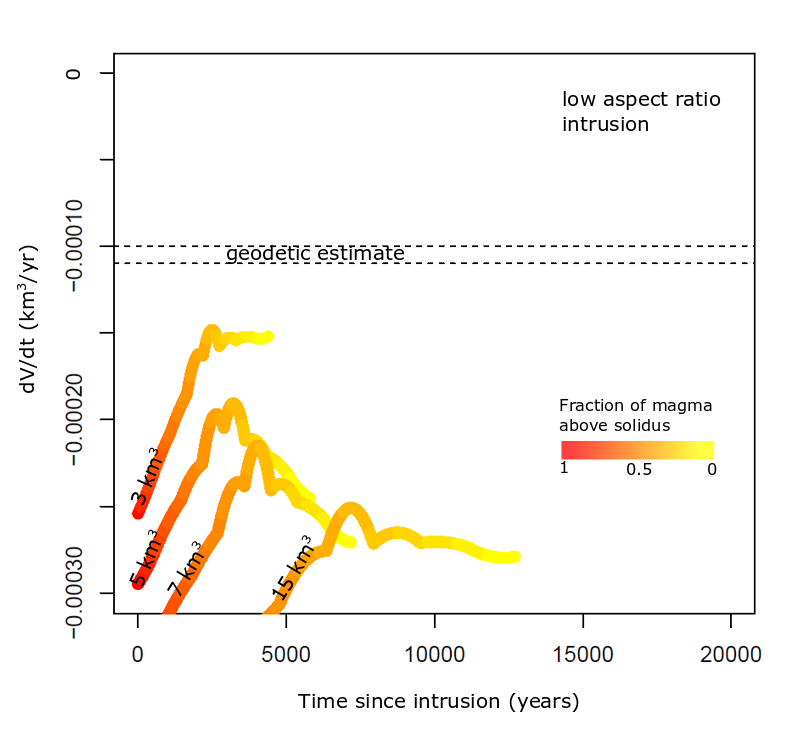


**Figure S4.** Thermal-petrological modelling of volume change rates for low aspect ratio of the reservoir. The volumetric change rate dV/dt is plotted as function of time since the onset of the intrusion. The curves are labelled according to their initial intrusion volume of 3.1, 5.0, 7.6 and 15.3 km^3^ with aspect ratio of ~0.2. Color coding identify the fraction of magma above solidus during cooling of the intrusions. The dashed line is the deflation rate calculated by inversion of geodetic data. No match could be obtained between the petrological and geodetic estimate for low aspect ratio intrusions.


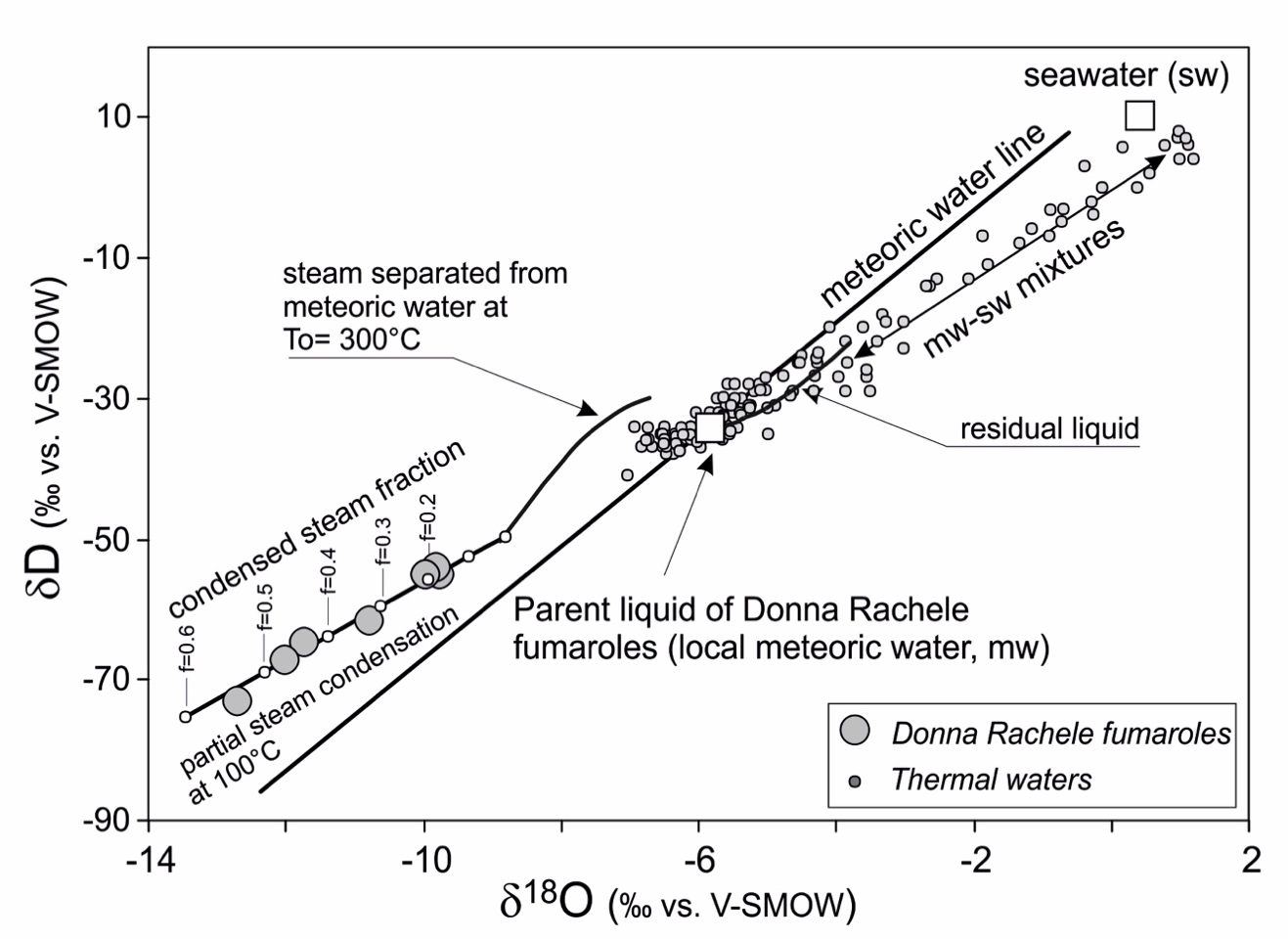


**Figure S5.** δD vs δ^18^O diagram of the thermal manifestation of Ischia. The fumaroles of Donna Rachele are compatible with (i) the boiling at high temperature (300°C) of a parent liquid of meteoric origin and (ii) the shallow partial condensation (at 100°C) of the original steam. All the thermal waters of the island are a mixture of the meteoric component and a thermal component of marine origin (data from Caliro et al., 1999; Di Napoli et al., 2009). The figure shows the absence of a significant amount of magmatic water whose isotopic composition is characterized by a much heavier oxygen (δ^18^O typically of + 10 ± 3 ‰) (Giggenbach, 1992). Data cannot, however, exclude the presence of a low proportion of magmatic water (e.g. < 5%).


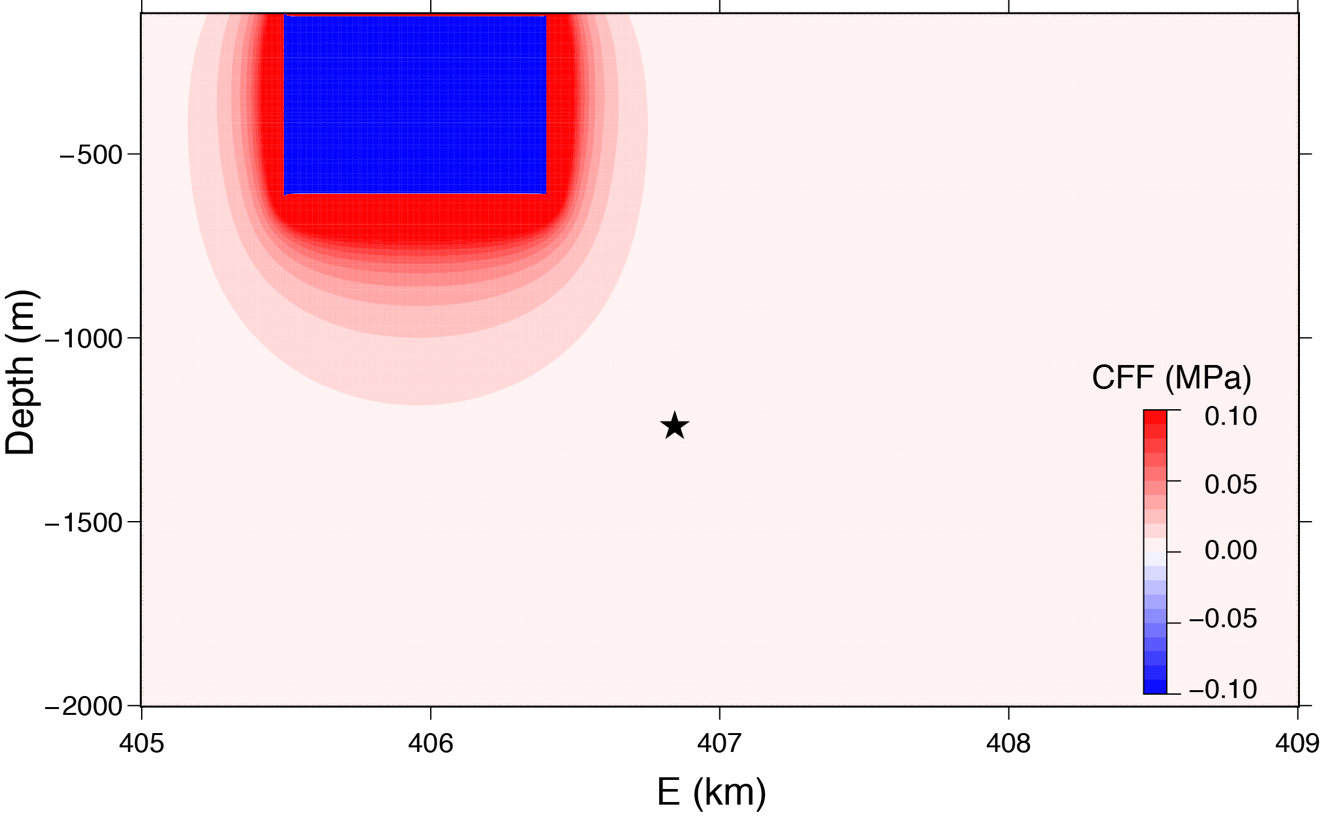


Figure S6. Coulomb Failure Function (CFF) results. The CFF is computed on a fault plane coincident with the modelled fault (Harris, 1998). East is in UTM projection, zone 33. The star is the hypocenter of the 2017 Casamicciola earthquake.

Table S1. Altimetric data and relative errors of the leveling measurements carried out between 1984 and 2010. (xls file). (File uploaded separately).

Table S2. Mean values of the parameters of the deformation sources at Ischia, constrained by geodetic data inversion.

| Model | E^a^ (km) | N^a^ (km) | Depth^a^ (km) | Dimension (m) | Intensity |
| --- | --- | --- | --- | --- | --- |
| Sill | 406.9 ± 1.5 | 4508.6 ± 1.5 | 2.2 ± 0.2 | 2100 ± 70  *radius* | -1.05 ± 0.07  *ΔV (10^5^ m^3^ yr^-1^) ^b^* |
| Fault | 405.5 | 4510.9 ± 0.1 | 0.12 ± 0.03 | 910 ± 20  *length* | 3.1 ± 0.4  *dip-slip (cm yr^-1^)* |

^a^ Easting and Northing are in UTM projection, zone 33. The position parameters for the fault are referred to the top-left corner.

^b^ Volume change rate not inverted but derived from its radius and potency (see maintext).

Table S3. Composition^a^ of Donna Rachele fumaroles.

| Sample | Date | T°C | H_2_O | CO_2_ | H_2_S | Ar | N_2_ | CH_4_ | H_2_ | He | CO | δ^18^O | δD |
| --- | --- | --- | --- | --- | --- | --- | --- | --- | --- | --- | --- | --- | --- |
| ISDR1B | 18/10/2000 | 99.2 | 997000 | 3000 | 30.0 | 0.10 | 11.5 | 0.182 | 16.6 | 0.090 | 0.0094 | -10.0 | -55.0 |
| ISDR1A | 18/10/2000 | 99.2 | 996000 | 3470 | 32.2 | 0.11 | 12.2 | 0.224 | 18.3 | 0.094 | 0.0109 | -10.0 | -55.0 |
| ISDR2 | 18/10/2000 | 99.2 | 996000 | 4390 | 49.0 | 0.36 | 36.8 | 0.317 | 23.6 | 0.103 | 0.0100 | -11.8 | -64.6 |
| ISDR1 | 03/05/2001 | 98.1 | 997000 | 2860 | 29.5 | 2.77 | 248.0 | 0.140 | 10.4 | 0.052 | 0.0065 | -9.8 | -55.1 |
| ISDR2 | 03/05/2001 | 97.4 | 996000 | 3040 | 39.4 | 5.39 | 473.0 | 0.221 | 14.3 | 0.061 | 0.0135 | -10.8 | -61.5 |
| ISDR3 | 03/05/2001 | 97.4 | 998000 | 2370 | 43.0 | 0.07 | 8.6 | 0.180 | 10.2 | 0.031 | 0.0085 | -12.7 | -73.0 |
| ISDR1 | 14/01/2002 | 99.4 | 997000 | 2910 | 20.9 | 0.38 | 29.8 | 0.181 | 12.6 | 0.061 | 0.0024 | -9.8 | -53.8 |
| ISDR2 | 14/01/2002 | 98.7 | 996000 | 3730 | 23.3 | 0.57 | 47.0 | 0.255 | 15.8 | 0.066 | 0.0029 | -12.0 | -67.2 |

^a^ Chemical concentrations (Chiodini et al., 2004) are expressed in μmol mol^-1^, oxygen, hydrogen isotopic compositions of steam condensed are reported in delta notation per mil vs. V-SMOW.

Bibliography

Caliro, S., Panichi, C., & Stanzione, D. (1999). Variation in the total dissolved carbon isotope composition of thermal waters of the Island of Ischia (Italy) and its implications for volcanic surveillance. *Journal of Volcanology and Geothermal Research, 90*(3-4), 219-240.

Chiodini, G., Avino, R., Brombach, T., Caliro, S., Cardellini, C., De Vita, S., et al. (2004). Fumarolic and diffuse soil degassing west of Mount Epomeo, Ischia, Italy. *Journal of Volcanology and Geothermal Research, 133*(1-4), 291-309.

Di Napoli, R., Aiuppa, A., Bellomo, S., Brusca, L., D'Alessandro, W., Candela, E. G., et al. (2009). A model for Ischia hydrothermal system: Evidences from the chemistry of thermal groundwaters. *Journal of Volcanology and Geothermal Research, 186*(3-4), 133-159.

Giggenbach, W. F. (1992). Isotopic Shifts in Waters from Geothermal and Volcanic Systems Along Convergent Plate Boundaries and Their Origin. *Earth and Planetary Science Letters, 113*(4), 495-510.

Harris, R. A. (1998). Introduction to special section: Stress triggers, stress shadows, and implications for seismic hazard. *Journal of Geophysical Research-Solid Earth, 103*(B10), 24347-24358.
